# Acanthocytes Identified in Huntington’s Disease

**DOI:** 10.3389/fnins.2022.913401

**Published:** 2022-06-06

**Authors:** Yueyi Yu, Yuanyuan Lu, Fen Wang, Yan Lu, Beijia Xie, Xiaosheng Meng, Yi Tang

**Affiliations:** ^1^Innovation Center for Neurological Disorders, Department of Neurology, Xuanwu Hospital, Capital Medical University, Beijing, China; ^2^Department of Clinical Medicine, Capital Medical University, Beijing, China

**Keywords:** Huntington’s disease (HD), acanthocytes, movement disorder, microscopy electron scanning, pathology

## Abstract

**Background:**

Neuroacanthocytosis (NA) and Huntington’s disease (HD) are neurodegenerative conditions that share clinical symptoms and imaging findings, despite their distinct genetic etiologies. Usually, the presence of acanthocytes can help narrow the differential diagnosis of a familial choreiform disorder, as the diagnosis of NA syndrome is supported by the presence of acanthocytes in peripheral blood. In this study, we demonstrate four patients who present with HD and acanthocytosis.

**Methods:**

We retrieved the data of 40 HD patients with fresh peripheral blood screened for erythrocytes in our hospital from 2014 to 2022. Of these 40 patients, four patients with acanthocytes were recruited for this study. Patients’ investigations included clinical and laboratory studies, *HTT* gene sequencing, and whole-exome sequencing. Fresh peripheral blood was screened for erythrocytes by scanning electron microscopy.

**Results:**

The four adult patients were Han Chinese and unrelated. The age ranged from 45 to 61 years, with a disease duration of 4–10 years. The main neurological features at diagnosis included progressive involuntary movements, psychiatric changes, and dementia. Genetic analysis showed an expansion at the *HTT* gene. The mean proportion of acanthocytes was mild (6–10%) elevated in patient one and high (>20%) elevated in patients 2–4 by scanning electron microscopy examination.

**Conclusion:**

Our study illustrates that HD can combine with acanthocytosis, which may expand the clinical phenotype. Even though the primary gene defect appears to be predominately directed at the brain, a peripheral defect can be seen in HD. Our study highlights the complexity and diversity of HD.

## Introduction

Huntington’s disease (HD) is a fatal, autosomal dominantly inherited neurodegenerative disorder that classically presents with movement disorders, mainly chorea, cognitive decline, and psychiatric symptoms (Margolis and Ross, 2003). HD is the commonest inherited cause of chorea, which is caused by variably expanded CAG trinucleotide repeats in exon 1 of the *huntingtin* (*HTT*) gene on chromosome 4p16.3 (Moily et al., 2014). An expanded allele (beyond a threshold of 35 units) through a deleterious gain-of-function mechanism leads to neuronal dysfunction and neurodegeneration. The first symptoms usually appear around the age of 40, with a life expectancy of around 18 years after the appearance of the first motor symptoms. Occasional cases with symptoms and signs suggestive of HD do not show an expansion mutation in the *HTT* gene. Different HD-like disorders, namely Huntington disease-like 1 (HDL1), Huntington disease-like 2 (HDL2), and spinocerebellar Ataxia Type 17 (SCA17) have been recognized with different underlying genetic causes (Schneider and Bird, 2016).

Neuroacanthocytosis (NA) syndromes are a group of rare diseases characterized by neurological disorders and misshaped spiky red blood cells (acanthocytes), with X-linked McLeod syndrome (MLS) and autosomal recessive Chorea-Acanthocytosis (ChAc) as the two best delineated conditions (Jung et al., 2011). NA is also found in a smaller percentage of cases with pantothenate kinase-associated neurodegeneration (PKAN), HDL2, and several inherited disorders of lipoprotein metabolism, namely abetalipoproteinemia (Bassen-Kornzweig syndrome) and hypobetalipoproteinemia leading to vitamin E malabsorption (Hayflick et al., 2003; Walker et al., 2003; Jung et al., 2011).

HD and NA are two of the genetic causes of chorea. It is sometimes difficult to distinguish them clinically because they have similar clinical symptoms and imaging findings. Usually, the presence of acanthocytes can help narrow the differential diagnosis of a familial choreiform disorder, as the diagnosis of NA syndrome is supported by the presence of acanthocytes in peripheral blood (Walker, 2007). In this article, we describe four unrelated patients who present with HD and acanthocytosis, a combination that has not been previously reported.

## Methods

### Patients

We retrieved the data of 40 HD patients with fresh peripheral blood screened for erythrocytes in our hospital from 2014 to 2022. Of these 40 patients, four patients with acanthocytes were recruited for this study. The expanded number of CAG repeats in HTT identified by direct sequencing and its correlation with the patients’ clinical symptoms confirmed the diagnosis of HD. Clinical data were extracted from patient records, including age at the first visit, age at onset, gender, family history, past history, clinical symptoms, neurological findings at the time of diagnosis, laboratory tests, and brain magnetic resonance imaging (MRI) findings at the time of diagnosis.

All patients were informed about this study, and written informed consent was obtained from every patient or patient’s legal guardian. This study was performed in accordance with the ethical standards laid down in the 1964 Declaration of Helsinki and its later amendments, and received approval from the ethics committee of Xuanwu Hospital, Capital Medical University.

### Scanning Electron Microscopy Analysis

In our patients, fresh peripheral blood was screened for erythrocytes by scanning electron microscopy (SEM) according to previous studies (Ciccoli et al., 2013). Briefly, aliquots of red blood cell suspensions were gently pelleted by centrifugation and fixed in 2.5% glutaraldehyde and 4% paraformaldehyde. After washing, the cells were dehydrated in 50–100% of graded series of ethanol, dried critical point, coated with a conducting material, and imaged with SEM. A patient with Wilson’s disease and a patient with peripheral facial neuritis were tested as controls at the same time. A board-certified clinical pathologist independently determined the erythrocytes in a blinded manner. Contracted red cells with a number of irregularly spaced thorny surface projections are defined as acanthocytes. The counts of acanthocytes within 3% were considered to be within the normal range. The mean proportion of acanthocytes in each patient was counted and divided into three classes: mild (6–10%), moderate (10–20%), and high (>20%).

### Genetic Analysis

Genomic DNA was extracted from peripheral EDTA blood using a DNA extraction kit. CAG triplet repeats of the *HTT* gene were identified by capillary electrophoresis and sequencing methods (Dong et al., 2013). In brief, a pair of primers (F:5′-CAGAGCCCCATTCATTGCC-3′, R: 5′-TGAGGAAGCTGAGGAGGC-3′) was used to amplify a DNA fragment in exon 1 in the CAG polymorphic region of the *HTT* gene under standard conditions. Polymerase chain reaction products were resolved by electrophoresis in an 8% polyacrylamide gel at 27 V/cm at 55°C for 3 h to determine the primary results. Then the length of CAG repeats was determined by Sanger sequencing. Furthermore, WES analysis was conducted on our patients. The target region of each sample had a sequencing depth greater than 100×, and the proportion of bases with a 20-fold depth of sequencing was greater than 97.10%.

## Results

### Patients

The main clinical features of the four patients with acanthocytes are summarized in Table 1. The four adult patients were Han Chinese and unrelated. The age ranged from 45 to 61 years, with a disease duration of 4–10 years. The four patients had rapid involuntary movements, three patients with psychiatric symptoms, and two patients with dementia. Their clinical symptoms are described below:

**TABLE 1:** Main clinical features of the patients included in this study.

Patient number	Sex	Age	Age of onset	Neurologic features	Family history	CAG repeats	Acanthocytes
P1	Male	61	57	Chorea, psychiatric changes, and dementia	+	42/16	9.3%
P2	Male	55	45	Chorea, personality changes, dysarthria, and ataxia	+	37/20	57.1%
P3	Female	45	35	Chorea, dementia, dysarthria, and ataxia	+	47/16	36.0%
P4	Female	56	52	Chorea and psychiatric changes	−	41/20	52.7%

#### Patient 1

A 61-year-old man presented with a 4-year history of rapid involuntary movements of the head and shoulders, then gradually progressed to the limbs. According to his relatives, he had also shown irritability, persecutory delusions, and cognitive problems. On neurological examination, there were choreic movements of the head, shoulders, and limbs. He presented with limb and trunk ataxia. Cognitive status was also impaired as assessed by neuropsychiatric questionnaires. Family history revealed that other family members also had a movement disorder. His 66-year-old sister had involuntary movements that started at the age of 60 years old. Additionally, his father’s sister presented cognitive decline and involuntary movements when she was 60 years old and died 15 years later without a definite diagnosis.

#### Patient 2

A 55-year-old man presented a 10-year history of rapid involuntary movement. The movements began first in his head and shoulders and later spread to his limbs, facial muscles, and tongue. Personality changes and slurred speech had been noted 2 years ago. On physical examination, prominent choreic movements of the limbs, head, shoulders, face, and tongue were present. He displayed dysarthria and ataxia of the limbs and trunk. Family history was significant for his mother and his three siblings with involuntary movements.

#### Patient 3

A 45-year-old woman presented a 10-year history of cognitive decline. At 40 years of age, she developed orofacial dyskinesia, slurred speech, and rapid involuntary movements in the limbs. The movements occurred during wakefulness and were absent in sleep. Neurological examination showed dysarthria and choreic movements. She presented with ataxia of the limbs and trunk, hypotonia, and hyperreflexia. Neuropsychometric tests revealed moderate dementia. She had a three-generation family history consistent with an autosomal dominantly inherited disease. Her maternal grandma, her mother, and her maternal uncle also had a movement disorder.

#### Patient 4

A 56-year-old woman presented a 4-year history of rapid involuntary movements in the trunk and limbs. According to the patient’s relatives, she had low mood, sleep disturbance, suicidal ideation, and slurred speech over the past 2 years. The psychiatric manifestations had preceded the involuntary movements. On physical examination, choreic movements of the trunk and limbs were present. There was no history of any abnormal movements, psychiatric manifestations, and no other members were affected in the family.

In our patients, the following tests were unremarkable: full blood count, hemoglobin, erythrocyte sedimentation rate, serum electrolytes, liver function, albumin, creatine phosphokinase, lipid metabolism, renal function, vitamins, and thyroid function. None of our patients had anorexia or used statins. The Brain MRI scan performed in all our patients demonstrated atrophy of the caudate nucleus and cortical atrophy.

### Scanning Electron Microscopy Analysis

**FIGURE 1:**
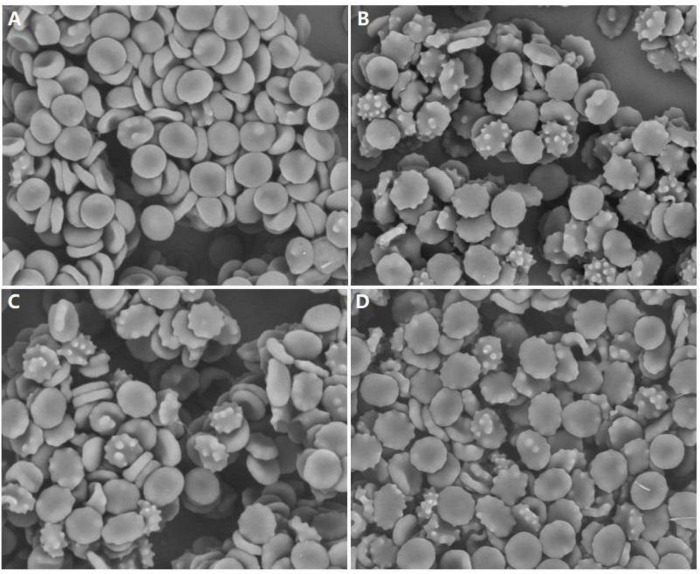
Examination of peripheral blood of our patients by scanning electron microscopy showing acanthocytosis. **(A)** Patient 1, **(B)** Patient 2, **(C)** Patient 3, **(D)** Patient 4, magnification: ×1800.

Acanthocytes were examined and counted in all our patients by SEM (Figure 1). The mean proportion of acanthocytes was mild (6–10%) in patient 1 and high (>20%) in patients 2–4. The controls did not have acanthocytes.

### Genetic Analysis

Genetic analysis showed an expansion in the *HTT* gene, with the CAG repeat number 42 in patient one, 37 in patient two, 47 in patient three, and 41 in patient four in the expanded allele. No additional potentially pathogenic variant was detected by WES.

## Discussion

HD is a slowly progressive, autosomal dominant neurodegenerative disease. The diagnosis of genetically confirmed HD is based on a CAG expansion of 36 or more repeats in the *HTT* gene (Disease Collaborative Research Group, 1993). In our study, four unrelated patients presented with a movement disorder, and genetic studies revealed expanded CAG trinucleotide repeats in the *HTT* gene, which is consistent with the diagnosis of HD.

Acanthocytosis refers to the transformation of the normal biconcave disk erythrocyte into one with a few irregularly shaped external projections distributed unevenly on its membrane surface. When examining for acanthocytes, it is important to avoid false positives, and up to 3% of acanthocytes are considered to be within the normal range in adult peripheral blood (Rampoldi et al., 2002). In our study, the experimental artifacts would not have developed because the acanthocytes are validated using a well-established and definitive methodology (Ciccoli et al., 2013), as fresh erythrocytes were promptly fixed in glutaraldehyde paraformaldehyde to preserve their morphology and then analyzed by SEM. All our HD patients had acanthocytes above the normal range. Considering the possibility that acanthocytosis may vary over time and may appear late during the disease, as has been reported in NA (Sorrentino et al., 1999), monitoring the peripheral blood of the patients will be necessary.

Given that the circumstances of genetically confirmed NA co-existing with *HTT* expansion have been reported (Murphy et al., 2018), it is important to rule out the co-existence of another choreiform disorder. Patients with HD present with cognitive impairment, behavioral dysfunction, and movement disorders. Patients with NA have additional symptoms, including orofacial self-mutilation, dyskinesia, seizures, amyotrophy, and peripheral neuropathy. In our patients, these common features of NA were absent. Combined with the pattern of inheritance and the WES result, NA was ruled out. We also excluded the acquired causes of acanthocytosis, including malnutrition, severe hepatic dysfunction, hypothyroidism, myelodysplasia, vitamin deficiency, and statin use. Our study illustrates that HD can combine with acanthocytosis. Therefore, when facing a choreiform disorder the *HTT* gene should always be performed.

Movement disorders associated with acanthocytosis include a variety of diseases. In NA, the proportion of acanthocytes is highly variable, usually between 5 and 50%, and does not seem to be correlated with the severity of the disease (Rampoldi et al., 2002). The presence of acanthocytes has usually been considered an important diagnostic marker of NA. However, sometimes the search for acanthocytes in NA can be misleading, as acanthocytes can appear late during the disease and can be absent in NA (Sorrentino et al., 1999). Furthermore, systemic diseases have to be considered, as is occasionally seen in malnutrition, severe hepatic dysfunction, thyroid disorders, hepatic encephalopathy, mitochondrial disease, and others.

Acanthocytosis occurs because of ultrastructural abnormalities of the erythrocyte membranous skeleton, which appears to be the result of disorganized membrane-cytoskeleton interactions due to defective endosomal trafficking and sorting, autophagic flux, and autolysosomal degradation in late-stage erythropoiesis (Prohaska et al., 2012; Lupo et al., 2016). However, the cause of both red blood cell and nervous system dysfunction remains largely unknown. It is hypothesized that an unidentified common pathway is responsible for both the altered red cell morphology and neurodegeneration, despite the distinct primary genetic cause (Jung et al., 2011).

HD is caused by a dominantly inherited CAG trinucleotide repeat expansion, which results in the production of a mutant huntingtin protein with an abnormally long polyglutamine repeat (Walker, 2007). The polyglutamine expansion causes a conformational change in *huntingtin*, leading to intracellular aggregates and neuronal dysfunction. Several molecular pathways have been implicated in the process of neurodegeneration involved in HD, which include protein clearance, protein-protein interaction, mitochondrial function, axonal trafficking, N-methyl-D-aspartate receptor activation, gene transcription, and posttranslational modification. Although the central nervous system is predominately affected in HD, growing evidence has shown substantial deficits in peripheral tissues due to the toxicity of mutant huntingtin proteins (Sassone et al., 2009). Cardiac dysfunction, muscle tissue loss, endocrine dysfunction, and blood tissue abnormalities have been described in HD patients.

Peripheral blood studies showed that mutant *HTT* induces a wide range of abnormalities in many types of blood cells, including lymphocytes, monocytes, macrophages, platelets, and erythrocytes (Chen et al., 2007; Sassone et al., 2009). The reduced activities of Cu/Zn-superoxide dismutase (Cu/Zn-SOD) and glutathione peroxidase (GPx) in the erythrocytes of HD patients were reported by Chen CM et al., indicating oxidative damage in erythrocytes (Chen et al., 2007). Markesbury et al. studied unmanipulated erythrocytes from HD patients under EM, which disclosed an increased number of stomatocytes in HD (Markesbery and Butterfield, 1977). Acanthocytosis and stomatocytosis are associated with defects of membrane proteins. According to the bilayer hypothesis, the shape of the erythrocytes reflects the ratio of the surface areas of the two hemileaflets of the lipid bilayer. The preferential expansion of the outer leaflet leads to acanthocytosis, whereas expansion of the inner lipid bilayer produces stomatocytosis. Our present study adds to evidence that erythrocyte membrane can be affected in HD. It was reported that an increase in the erythrocyte membrane-bound Na^+^K^+^-ATPase, an increase in chlorine ion transport, and a decrease in plasma GABA levels may associate with the alternation of erythrocytes membranes in HD (Beverstock, 1984). Unfortunately, most of these results have not been further investigated in the recent 40 years. Currently, there is still much ambiguity about the erythrocytes abnormality in HD at the molecular level. Further studies to elucidate the underlying mechanism will be necessary.

Our work has several limitations. First, all subjects in the present study were ethnic Chinese. The possible findings in other ethnicities need to be explored. Second, light microscopy was not performed in our patients. Because examining blood smears by light microscopy had limited sensitivity in our experience as well as in previous studies (Feinberg et al., 1991), fresh peripheral blood was screened for erythrocytes by scanning electron microscopy. Finally, we lack blood samples to perform further red cell membrane examination that could provide some insight into the potential molecular mechanisms in our patients.

## Conclusion

In conclusion, our study provides evidence that HD can combine with acanthocytosis, which may expand the clinical phenotype. Even though the primary gene defect appears to be predominately directed at the brain, a peripheral defect can be seen in HD. Our study highlights the complexity and diversity of HD. A meticulous clinical evaluation, laboratory tests, and genetic study are essential to make the final diagnosis.

## Data Availability Statement

The raw data supporting the conclusions of this article will be made available by the authors, without undue reservation.

## Ethics Statement

The studies involving human participants were reviewed and approved by the Ethics Committee of Xuanwu Hospital, Capital Medical University. The patients/participants provided their written informed consent to participate in this study.

## Author Contributions

YY and YT conceived the design of the study. YuL wrote the first draft of the manuscript. YY, BX, and XM contributed to the cases diagnosis and data collection. FW and YaL help revised the manuscript. All authors read and approved the final version of the manuscript.

## Conflict of Interest

The authors declare that the research was conducted in the absence of any commercial or financial relationships that could be construed as a potential conflict of interest.

## Publisher’s Note

All claims expressed in this article are solely those of the authors and do not necessarily represent those of their affiliated organizations, or those of the publisher, the editors and the reviewers. Any product that may be evaluated in this article, or claim that may be made by its manufacturer, is not guaranteed or endorsed by the publisher.
